# Efficient immune reconstitution in HIV+ naïve patients (pts) starting a first lopinavir/ritonavir-containing regimen with low CD4 counts

**DOI:** 10.1186/1758-2652-13-S4-P17

**Published:** 2010-11-08

**Authors:** E Merlini, E Sinigaglia, G Carpani, T Bini, A d'Arminio Monforte, GC Marchetti

**Affiliations:** 1 "San Paolo" Hospital, Clinic of Infectious Diseases, Milan, Italy; 2 "San Paolo" Hospital, Blood Transfusion Center, Milan, Italy; 3 "San Paolo Hospital", Clinic of Infectious and Tropical Diseases, Milan, Italy

## Purpose of the study

Investigate immune restoration profile, T-cell activation and microbial translocation in HIV+ naïve pts starting a first LPV/r-containing regimen with low CD4.

## Methods

40 HIV+ antiretroviral-naive pts starting a first tenofovir/emtricitabine + LPV/r-containing ART with CD4 <350 (20 Late Presenters —LPs, CD4 <100/µL and 20 Non-Late Presenters —NLPs, CD4, 200—350/µL) were followed for 12 months (T12). Microbial translocation (MT) by plasma lipopolysaccharide (LPS) and sCD14 (LAL assay and ELISA), CD38+CD8, CD45R0+38+CD8, CD127+CD4/CD8 (flow cytometry), and plasma IL-7 (ELISA) were tested at T0 and T12. T0 and T12 differences were analyzed by Mann Whitney U test.

## Summary of results

At T12, all 40 HIV+ pts displayed a significant CD4 rise, HIV viremia reduction (p=.0006; p<.0001, respectively) and a decrease in activated CD38+CD8 (p<.0001), with a trend to an increase in CD127+CD8 (p=.07). By T12, both LPs and NLPs displayed a significant CD4 increase (LPs: p=.0001; NLPs: p=.001), with LPs maintaining significantly lower CD4 at T12 (p=.0001). At T12, NLPs and LPs displayed a significant reduction in CD38+CD8+ (p=.009; p=.018, respectively); only NLPs displayed a decreasing trend in terminally-differentiated CD45R0+CD38+CD8 (p=.077). Compared to LPs, NLPs featured higher CD127+CD4 proportions at all timepoints (T0, p=.0001; T12, p=.001), with a significant increase in CD127+CD8 by T12 (p=.012), whereas no changes were seen in LPs. NLPs also displayed a significant rise in circulating IL-7 (p=.049), whereas LPs showed a decreasing trend (p=.074). At T0, NLPs showed higher levels of MT markers (LPS: p=.01; sCD14: p=.007). By T12, only NLPs displayed a significant reduction in LPS (p=.022) and in sCD14 (p=.005), whereas no changes were shown in LPs.

## Conclusions

In HIV+ antiretroviral-naive pts with low CD4, LPV/r-containing regimens resulted in adequate immune reconstitution and restoration of the IL-7/IL-7R system. Interestingly, microbial translocation was efficiently controlled only in patients with less advanced HIV infection. However, LPV/r-based treatment resulted in a significant reduction of peripheral T-cell activation also in patients with late presentation. Given that T-cell activation is predictive of disease progression, our data advocate the efficacy of LPV/r regimens in broad immune reconstitution in HIV-infected pts with advanced infection.

